# Prof. W. D. Miller.—A Reception

**Published:** 1890-11

**Authors:** 


### Prof. W. D. Miller.—A Reception.

Immediately after the adjournment of the recent meeting of the International Medical Congress at Berlin, Prof. W. D. Miller left that city for a brief visit to his native country—the United States.

This was gratifying indeed to the Dentists of this country, at least, so far as they were able to meet him. No one from any quarter could have met with a more cordial reception than Dr. Miller.

He has done a great work for dental science, and is still doing. The profession owes him a debt that it can never pay.

On the 27th of September it was the privilege of the Odontological Society of Cincinnati to have a visit from Dr. Miller, and in the evening a reception and entertainment in the very pleasant parlors of the University Club. After general social converse for an hour, the guest, and twenty three members of the Society were seated at a well appointed table which had been prepared under the skilful direction of Mr. Bruno Bolz, the Steward.

The following members were present, namely: Drs. F. H. Hunter, President of the Odontological Society, C. M. Wright, O. N. Heise, Wm. M. Williams, J. R. Callahan, J. Taft, J. S. Cassidy, E. G. Betty, H. M. Fletcher, A. G. Rose, H. A. Smith, F. W. Sage, J. I. Taylor, G. Mollenaux, H. C. Matlock, R. E. Taylor, C. H. Martin, R. G. Poore, W. A. Betman, J. G. Cameron, D. W. Roudebush, H. T. Smith and J. Leslie.

After the good things of the table had been disposed of, Dr. Hunter in a brief, well-worded address, referred very appropriately to the position attained by the guest, and referred to the work he had accomplished; after which, Dr. Miller in a brief address gave some account of the work he had been enabled to accomplish, and the great difficulties he had encountered, and the persistent labor that had been required to attain success; and notwithstanding the work he has accomplished in another land and the opportunities that have there been afforded him, he expressed a strong preference for his native country, and especially for Ohio as his native state. After this, remarks appropriate to the occasion on various lines of thought were made by quite a number of those present, in all of which a very high appreciation of that which Dr. Miller has done was expressed.

As a result of Dr. Miller's advanced scientific work, he has attained a position accorded to no other man of foreign birth in the country of his present residence, and indeed, so far as we can now call to mind, in no European country has an American ever attained so high a position as has Dr. Miller. The profession is highly pleased to acknowledge him as an American, and the dental profession of Ohio especially so. May nothing ever come in the way of his progress and the fulfilment of his highest desire.

